# Engineering and Biological Mechanisms of Microalgal CO_2_ Fixation: A Review from Molecular Regulation to System Optimization

**DOI:** 10.3390/microorganisms14050999

**Published:** 2026-04-29

**Authors:** Zhongliang Sun, Weixian Chen, Yu Xie, Shoukai Guo, Liqin Sun, Qiang Wang

**Affiliations:** 1 School of Life Sciences, Yantai University, Yantai 264005, China; 2 Institute of Systems, Molecular and Integrative Biology, University of Liverpool, Liverpool L69 7ZX, UK; 3 State Key Laboratory of Crop Stress Adaptation and Improvement, School of Life Sciences, Henan University, Kaifeng 475004, China

**Keywords:** microalgal CO_2_ fixation, carbon concentrating mechanisms, Rubisco enzyme, carbonic anhydrase, multiscale metabolic regulation, intelligent photobioreactor engineering

## Abstract

Microalgae are among the most efficient photosynthetic organisms on Earth, and their capacity for CO_2_ fixation directly links the global carbon cycle with green energy conversion, positioning them as strategic biological platforms for achieving carbon neutrality. This review provides a comprehensive and multiscale synthesis of the engineering and biological mechanisms underlying microalgal CO_2_ fixation, integrating perspectives from gas–liquid mass transfer, CO_2_ assimilation pathways, key enzymatic systems, metabolic regulation, and environmental control. From an engineering standpoint, we analyze the limitations governing CO_2_ transfer from the gas phase to the aqueous phase and critically evaluate intensification strategies aimed at enhancing inorganic carbon availability in cultivation systems. At the biological and biochemical levels, we dissect carbon concentrating mechanisms (CCMs), including C_4_-like pathways, and elucidate the structural organization, regulatory properties, and functional coordination of Rubisco and carbonic anhydrase systems. Particular emphasis is placed on the coupling between enzyme-level regulation and metabolic flux redistribution, supported by insights from metabolic flux analysis and systems-level modeling, to establish theoretical and engineering foundations for improving carboxylation efficiency. Finally, we propose an integrated roadmap for the future development of microalgal CO_2_ fixation technologies, highlighting the convergence of synthetic biology, artificial intelligence, and systems engineering to achieve end-to-end optimization from molecular mechanisms to reactor-scale performance, while enabling the valorization of waste gas streams and circular carbon utilization. This review aims to provide a coherent theoretical framework and forward looking perspective for the development of efficient, intelligent, and sustainable microalgal CO_2_ fixation systems.

## 1. Introduction

As global climate change intensifies and carbon neutrality strategies are increasingly prioritized worldwide, microalgae have emerged as highly promising biological platforms for CO_2_ fixation due to their abundant diversity, rapid growth rates, and exceptional photosynthetic performance [1]. The photosynthetic efficiency of microalgae can be three to five times higher than that of terrestrial plants, and microalgal systems are estimated to contribute nearly 40% of global annual CO_2_ fixation [2]. More importantly, microalgal CO_2_ fixation not only directly reduces atmospheric CO_2_ concentrations but also enables the conversion of inorganic carbon into bioenergy carriers and high-value bioproducts, thereby forming a closed-loop “CO_2_ capture–conversion–utilization” framework [3]. In recent years, driven by the convergence of synthetic biology and process engineering, research on microalgal CO_2_ fixation has progressed from phenomenological observations toward systematic elucidation of molecular mechanisms and engineering applications [4].

Microalgal CO_2_ fixation is intrinsically a complex, multiscale process spanning physical mass transfer constraints and sophisticated biological regulation. From an engineering perspective, the efficient transfer of CO_2_ from the gas phase to the aqueous cellular environment represents a primary bottleneck limiting overall fixation efficiency [5]. From a biological perspective, microalgae have evolved diverse and highly efficient carbon assimilation strategies, including carbon concentrating mechanisms (CCMs), C_4_-like pathways, and multiple inorganic carbon uptake and utilization systems [6]. The coordinated operation of these processes, rather than the optimization of individual components in isolation, ultimately determines CO_2_ fixation performance. To systematically characterize the knowledge structure and evolving research frontiers in microalgal CO_2_ fixation, bibliometric data were retrieved from the Web of Science Core Collection (Supplementary Materials Part I and Figure S1). Guided by these insights, a mechanistic understanding of how mass transfer processes interact with intracellular carbon assimilation and regulatory networks is essential for rational system optimization.

In this review, we systematically synthesize advances in microalgal CO_2_ fixation from both engineering and biological perspectives. Particular emphasis is placed on two interrelated themes: (i) gas–liquid mass transfer processes and their intensification strategies, and (ii) carbon assimilation pathways, key enzymatic systems, and metabolic regulation. By integrating insights across these dimensions, this review aims to establish a coherent knowledge framework for understanding the mechanisms governing microalgal CO_2_ fixation and to provide strategic guidance for future technological innovation and system-level optimization.

## 2. Gas–Liquid Mass Transfer and Intensification Strategies in Microalgal CO_2_ Fixation

### 2.1. Fundamental Constraints of CO_2_ Gas–Liquid Mass Transfer in Microalgal Systems

In microalgal CO_2_ fixation processes, the first and often rate-limiting step is not intracellular carboxylation but the delivery of inorganic carbon from the gas phase into an aqueous medium where cells can access CO_2_(aq) and HCO_3_^−^. Importantly, CO_2_ transfer in photobioreactors cannot be treated as a simple physical absorption process: CO_2_ dissolution is coupled to rapid aqueous-phase speciation (CO_2_(aq)/HCO_3_^−^/CO_3_^2−^), meaning that pH dynamics, alkalinity, and stripping/degassing jointly shape the effective driving force and the net carbon balance [7]. Two coupled constraints dominate this upstream bottleneck. First, Henry’s law imposes a thermodynamic ceiling on dissolved CO_2_ concentration for a given gas-phase partial pressure, which becomes particularly restrictive under atmospheric or low-enrichment aeration conditions [5]. Second, the hydrodynamic renewal of the gas–liquid interface controls the thickness of the liquid-side boundary layer and is commonly captured by the volumetric mass-transfer coefficient *K_L,a_*. The gas–liquid mass transfer flux can be expressed as:

(1) $$N=K_{L,a}(C^{*}-C)$$

where *N* is the mass transfer flux, C* is the carbon dioxide saturation concentration, and *C* is the bulk liquid concentration. Inadequate mixing, short bubble residence times, and bubble coalescence can all depress effective *K_L,a_* and thus the net CO_2_ supply rate [5].

Unlike inert-gas absorption, CO_2_ transfer in algal cultures is inseparable from carbonate chemistry and biological uptake. Dissolved CO_2_ rapidly interconverts with HCO_3_^−^/CO_3_^2−^, making pH and alkalinity integral determinants of the “effective” driving force for absorption and the propensity for stripping/degassing [5]. Consequently, simply increasing aeration does not guarantee improved carbon capture: enhanced turbulence may accelerate both absorption and desorption, especially when photosynthetic uptake is insufficient to maintain undersaturation [5]. This coupling also complicates *K_L,a_* estimation and scale-up. CFD-based analysis in airlift photobioreactors shows that *K_L,a_* can be strongly non-uniform in space and that *K_L,a_* values inferred from standard dynamic gassing methods are sensitive to how CO_2_ depletion and aqueous-phase dissociation are represented-issues that intensify as reactor dimensions and hydrodynamic heterogeneity increase [8]. Complementing this, controlled experiments that simultaneously track gas- and liquid-phase carbon fluxes demonstrate that apparent CO_2_ transfer and growth can be tightly linked, and that the presence of cells can measurably affect gas–liquid transfer behavior, underscoring that “physical” mass transfer is dynamically coupled to biological sinks [5]. At the process level, these constraints translate into substantial carbon losses when CO_2_ delivery is poorly matched to cellular demand. Collectively, these studies indicate that overcoming the mass-transfer bottleneck requires treating CO_2_ supply as an integrated problem of interfacial transport, carbonate equilibria, and culture physiology—an essential prerequisite for realizing the downstream benefits of CCM–carbonic anhydrase–Rubisco coordination [5].

### 2.2. Engineering Strategies for Gas–Liquid Mass Transfer Enhancement in Photobioreactors

Conventional microalgal cultivation systems rely on a limited set of reactor architectures, including open raceway ponds and closed photobioreactors such as flat-panel, tubular, bubble column, airlift, and stirred-tank configurations. Although these systems differ in geometry and operation, their performance in CO_2_ fixation is ultimately constrained by how effectively each architecture renews the gas–liquid interface and sustains a favorable mass-transfer driving force [9]. Open raceway ponds offer low capital and operating costs but typically suffer from weak interfacial renewal and rapid CO_2_ escape, resulting in low effective carbon availability [9]. Closed photobioreactors alleviate some of these limitations, yet their advantages depend on the balance between achievable volumetric mass-transfer coefficients (*K_L,a_*), gas residence time, and energy input [10]. Table 1 summarizes representative gas–liquid mass transfer performances of conventional microalgal cultivation systems, highlighting large variations in *K_L,a_*, CO_2_ utilization, and technology maturity across reactor architectures.

Among closed systems, vertical bubble column reactors consistently exhibit higher *K_L,a_* values than airlift and stirred-tank designs under comparable aeration, reflecting enhanced gas holdup and interfacial renewal [12]. Airlift reactors improve circulation and reduce shear but often deliver intermediate mass-transfer performance, while stirred tanks provide strong mixing at the expense of higher energy dissipation and comparatively low CO_2_ transfer efficiency [10]. Flat-panel photobioreactors achieve superior light distribution and short diffusion distances; however, CO_2_ supply can still become limiting at high cell densities unless gas–liquid contact time is deliberately extended through structural or hydrodynamic modifications [13,14]. Tubular photobioreactors offer high surface-area-to-volume ratios but are prone to CO_2_ transfer limitations associated with short gas residence times and high circulation demand; integrating microbubble-based dissolution modules has been shown to substantially enhance *K_L,a_* and biomass productivity under simulated flue gas conditions [16]. Collectively, these comparisons demonstrate that reactor selection is fundamentally a choice of mass-transfer architecture, and that improvements in CO_2_ fixation performance require explicit engineering of interfacial area, renewal frequency, and energy efficiency rather than reliance on reactor geometry alone [9,17].

Building on conventional reactor hydrodynamics, the second lever is interfacial engineering, which involves increasing the gas–liquid contact area and extending the residence time of CO_2_. In *Chlorella pyrenoidosa*, systematically varying bubble size altered CO_2_ fixation and biomass synthesis and triggered measurable metabolic responses, indicating that “mass transfer design” propagates into cellular carbon allocation rather than only changing *K_L,a_* in a black-box manner [15]. Complementarily, CO_2_ nanobubbles were shown to improve dissolution and gas–liquid mass transfer behavior, while also stabilizing CO_2_ supply and pH under algal cultivation conditions; generation mode further shaped nanobubble density and transfer kinetics [18]. Together, these studies support the following design rule: bubble miniaturisation is not merely a tactic to intensify processes; it also alters the temporal profile of inorganic carbon exposure, which can alter physiology (e.g., carbon partitioning and the stress response) [15].

A third lever is to decouple carbon delivery from sparging losses via membranes. A liquid–liquid membrane contactor that transfers CO_2_ captured in solvent into a raceway pond achieved markedly higher CO_2_ utilization than air sparging, reaching up to 90% under optimized configurations, while maintaining comparable productivity to direct bubbling and lowering energy cost through controlled solvent delivery [19]. A study was conducted at the capture–delivery interface, which demonstrated a solvent absorption and non-porous PDMS hollow-fibre desorption concept. This concept avoids conventional solvent regeneration and gas compression, enabling carbon delivery that removes carbon limitation and increases volumetric productivity (reported up to 0.38 g L^−1^ d^−1^) [20].

Beyond hardware-based intensification, CO_2_ mass transfer can be strengthened by reaction/solubility engineering in the culture medium. The introduction of CO_2_ absorbents (e.g., alkanolamines) results in a chemical enhancement factor (β > 1) and the formation of carbamates that function as recyclable CO_2_ carriers, thereby serving as a slow-release inorganic carbon reservoir as cells consume CO_2_/HCO_3_^−^. This process leads to an increase in CO_2_ utilization (e.g., column systems improved from ~44.5% to ~76.1% under optimized dosing) while requiring careful biocompatibility control at high absorbent concentrations [21,22]. Exogenous carbonic anhydrase (CA) further couples absorption to conversion by accelerating CO_2_/HCO_3_^−^ interconversion and lowering liquid-phase inorganic carbon source, which sustains the gas–liquid driving force; because free CA is shear-labile in bubbling systems, immobilized CA (beads, electrospun fibers, CA-coated membranes) is favored for continuous operation [23,24].

### 2.3. Coupled Light–Mass Transfer Optimization and Emerging Intelligent Transport Strategies

Microalgal CO_2_ fixation at scale is increasingly constrained by the dynamic mismatch between CO_2_ delivery, light availability, and cellular uptake capacity. Consequently, the next wave of mass-transfer innovation is shifting from hardware-based intensification toward model-enabled, sensor-driven, closed-loop operation. Mechanistic models remain essential as priors for scale-up and process control. For example, tank-in-series and kinetic models have been applied to describe flue-gas CO_2_ utilization in bubble-column cultivation, providing a quantitative basis for evaluating gas-use efficiency under realistic operating constraints [25]. More recently, CFD-based frameworks have explicitly coupled hydrodynamics, CO_2_ dissolution, and growth responses to dissolved CO_2_ availability, enabling systematic diagnosis of mass-transfer limitations and objective comparison of reactor designs across operating regimes [26].

In parallel, artificial intelligence and machine learning (AI/ML) are emerging as the operational layer that translates high-frequency sensor data into actionable control decisions. An integrated tubular photobioreactor study demonstrated that Internet-of-Things sensors (pH, temperature, light intensity, electrical conductivity, flow rate, and dissolved oxygen), combined with non-invasive image-based biomass estimation, can feed ML models such as random forest and XGBoost to predict growth and automatically regulate pumping and illumination [27]. At the process level, hybrid neuro-fuzzy systems and evolutionary optimization algorithms have been applied to forecast CO_2_ fixation performance across strains and cultivation conditions, indicating that data-driven surrogates can effectively complement mechanistic models when parameterization is challenging [28]. Broader reviews further indicate that AI/ML applications are expanding from monitoring toward integrated optimization, fault detection, and advanced process control throughout the microalgae production chain [29].

## 3. Carbon Concentrating Mechanisms in Microalgae

While advanced mass-transfer strategies can substantially enhance CO_2_ availability in the culture medium, the ultimate fate of this carbon is determined by how efficiently microalgal cells capture, concentrate, and utilize inorganic carbon at the intracellular level. In aquatic environments, CO_2_ diffusion is substantially slower than in air, and dissolved inorganic carbon (DIC) predominantly exists as bicarbonate (HCO_3_^−^), rather than free CO_2_ (aq). Consequently, Rubisco often operates under stronger substrate limitation in water than in most terrestrial systems, particularly under low-CO_2_ conditions [30]. Rather than evolving fundamentally higher intrinsic catalytic efficiencies, algae and other photosynthetic microorganisms have repeatedly and independently developed carbon concentrating mechanisms (CCMs). The shared functional objective of CCMs is to establish a localized high-CO_2_/low-O_2_ microenvironment in the immediate vicinity of Rubisco, thereby alleviating kinetic constraints imposed by its relatively low turnover rate and susceptibility to oxygenation reactions [31]. Phylogenetic analyses reveal that CCMs have emerged multiple times across cyanobacteria, green algae, diatoms, and dinoflagellates, reflecting striking convergent evolution in response to inorganic carbon limitation [32].

### 3.1. Functional Types and Conceptual Frameworks of CCMs

**Figure 1:**
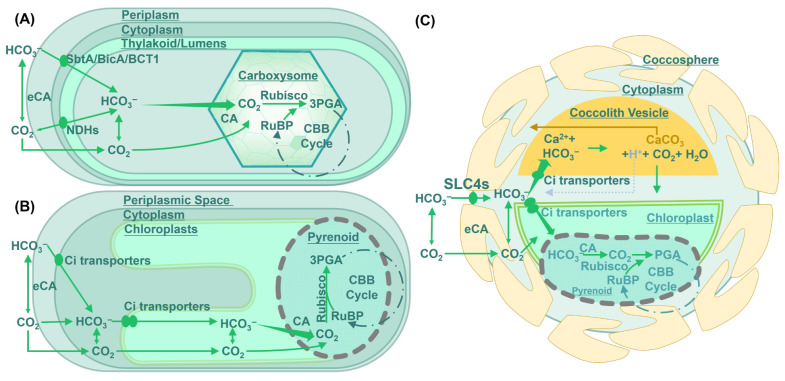
**Structural and functional diversity of microalgal CO_2_-concentrating mechanisms (CCMs).** (**A**) Cyanobacterial carboxysome-based CCM. (**B**) Eukaryotic pyrenoid-based CCM in green algae. (**C**) Biophysical CCM and calcification–photosynthesis coupling in the coccolithophore *Emiliania huxleyi*.

Despite the considerable variation in CCM architectures and metabolic pathways among photosynthetic lineages, microalgal CCMs can be broadly classified according to their modes of CO_2_ enrichment. The classification system distinguishes three principal categories: biophysical CCMs, biochemical (C_4_-like) CCMs, and hybrid or composite CCMs [32]. As demonstrated in Figure 1 and Table 2, the primary categories of CCMs can be identified.

### 3.2. Biophysical CCMs

Biophysical CCMs are among most extensively studied and most widely distributed form of carbon concentration systems, occurring in cyanobacteria and numerous eukaryotic microalgae. Their defining feature is the combination of active inorganic carbon uptake with spatial micro-compartmentalization, enabling localized CO_2_ levels around Rubisco [33]. A substantial fraction of global marine CO_2_ fixation is mediated by organisms harboring carbon-fixing micro-compartments, particularly cyanobacteria and pyrenoid-containing algae [35]. The core principle involves the generation of an intracellular inorganic carbon (Ci) pool through membrane transport systems, followed by spatially controlled conversion of HCO_3_^−^ to CO_2_ in proximity to Rubisco. This organization effectively reduced oxygenation reactions and carboxylation efficiency [36].

From structural and evolutionary perspectives, biophysical CCMs rely primarily on two distinct micro-compartments: carboxysomes in cyanobacteria and pyrenoids in eukaryotic algae [33]. Despite their structural differences, both systems comprise three functional modules: (1) Ci transport systems, (2) spatially controlled carbonic anhydrase (CA) activity, (3) localized enrichment of Rubisco with diffusion restrictive compartments [31,37,38]. These different functional types and conceptual frameworks of Biophysical CCMs were summarized and discussed in the Supplementary Materials Part II.

### 3.3. Biochemical (C_4_-like) CCMs

Classical C_4_ photosynthesis in vascular plants relies on spatial separation between mesophyll and bundle sheath cells, involves initial HCO_3_^−^ fixation by phosphoenolpyruvate carboxylase (PEPC) to form oxaloacetate (OAA), subsequent interconversion to malate or aspartate via malate dehydrogenase (MDH), decarboxylation by malic enzymes (ME) or PEP carboxykinase (PEPCK), and regeneration of phosphoenolpyruvate (PEP) through PPDK or PEPCK [39]. Microalgae lack tissue-level compartmentalization and were long assumed incapable C_4_ metabolism. However, accumulating evidence indicates the presence of C_4_-like biochemical CCMs in several algal lineages, providing a new conceptual framework for understanding enhanced carbon fixation under carbon-limited conditions [34].

In these systems, HCO_3_^−^ may be initially fixed by PEPC in the cytosol to form OAA, followed by conversion to malate or aspartate and transported into chloroplasts or mitochondria. Decarboxylation by NAD-ME, NADP-ME, or PEPCK locally releases CO_2_, potentially elevating CO_2_ concentrations near Rubisco. This process resembles a single-cell C_4_ cycle, in which metabolic compartmentalization partially substitutes for anatomical separation [40]. C_4_-like pathways are often induced under low CO_2_, high light, elevated photorespiratory pressure, or nutrient limitation [34].

Unlike canonical terrestrial C_4_ photosynthesis, sustained high-flux cyclic operation comparable to plant C_4_ systems has not been conclusively demonstrated in most microalgae. Diatoms, such as *Phaeodactylum tricornutum* and *Thalassiosira pseudonana*, exhibits pronounced upregulation of PEPC, PEPCK, and NADP-ME under low CO_2_ [41,42]. In green algae, *Chlorella vulgaris* and *Chlamydomonas reinhardtii* show PEPC activation and C_4_-acid cycling features [43]. Species of *Nannochloropsis* may harbor both structural and biochemical CCM components, suggesting more complex carbon concentration strategies, although definitive metabolic evidence remains limited and sometimes contradictory [44]. Many microalgae, even if they possess C_4_-like mechanisms, may contribute relatively little under natural conditions [34].

Overall, while most microalgae rely primarily on C_3_ photosynthesis, C_4_-like biochemical pathways may provide conditional advantages under carbon-poor environments or high-photorespiratory conditions. Their quantitative contribution under natural environments remains to be rigorously resolved. Artificial compartmentalization or modular introduction of selected C_4_ reactions may provide future strategies to synergize with native CCMs, although their feasibility and net benefit require rigorous experimental validation.

### 3.4. Hybrid or Composite Carbon Concentrating Mechanisms

In contrast to cyanobacteria, several eukaryotic microalgae with complex endosymbiotic origins exhibit more elaborate carbon concentrating strategies. These are often described as hybrid or composite CCMs, reflecting the integration of multiple CO_2_-enrichment mechanisms within a single dominant pathway [30,45]. Hybrid CCMs combine active bicarbonate uptake, auxiliary C_4_-like enzymatic reactions, and structural diffusion constraints imposed by multilayered plastid membranes [46]. In organisms derived from secondary or tertiary endosymbiosis, additional membranes and periplastidial compartments create inherent spatial organization that may facilitate coordinated carbon routing and retention [47].

#### 3.4.1. Functional Modules of Hybrid CCMs

Hybrid CCMs can be conceptualized as integrated assemblies of transport, catalytic, and structural modules. Active HCO_3_^−^ transporters located at the plasma membrane, chloroplast envelope, and thylakoid membranes establish an intracellular DIC reservoir. As discussed above for diatoms, SLC4-family transporters frequently represent central determinants of bicarbonate uptake [48].

In parallel, C_4_-like enzymes such as PEPC, MDH, ME, PEPCK may be differentially expressed under CO_2_ limitation, potentially contributing to localized CO_2_ release. However, the magnitude of their contribution to net carbon acquisition remains uncertain and may vary across taxa [41].

Finally, structural features, including multilayered plastid envelopes and internal membrane architectures, likely impose diffusion constrains that reduce CO_2_ leakage and shape intracellular carbon fluxes. Rather than functioning as discrete pathways, these components appear to operate as partially overlapping modules whose relative contributions shift in response to environmental conditions [49].

#### 3.4.2. Diatoms as a Paradigm of Hybrid CCMs

Diatoms are widely regarded as representative examples of hybrid CCMs organization. They possess efficient HCO_3_^−^ uptake systems dominated by SLC4-family transporters, enabling robust DIC accumulation from seawater under low CO_2_ conditions [50].

A defining feature of hybrid CCMs is their dynamic regulatory plasticity. Different modules can be differentially activated or attenuated in response to ambient CO_2_ availability, light intensity, nutrient limitation, or mixotrophic growth conditions. This flexibility allows microalgae to switch between alternative carbon acquisition strategies, thereby maintaining efficient carbon capture in highly variable marine environments [51].

#### 3.4.3. Current Limitations and Conceptual Implications

Despite increasing mechanistic insight, the internal coordination and quantitative flux distribution among hybrid CCMs components remain incompletely resolved. In particular, the functional significance of C_4_-like reactions remains contentious, with conflicting evidence regarding their role as dedicated CO_2_ pumps [51].

Most existing evidence is derived from transcriptomic profiling or enzyme activity assays, whereas direct subcellular flux measurements and stable isotope tracing remain comparatively scarce. Accordingly, hybrid CCMs are best interpreted as dynamic, modular assemblies rather than fixed canonical pathways. Future integration of multi-omics analyses, quantitative flux measurements, and mechanistic modeling will be essential for disentangling cooperative interactions among transport, catalytic, and structural modules. Such understanding will inform rational engineering strategies aimed at reconstructing or redesigning CCMs architectures for enhanced microalgal carbon capture.

In this context, hybrid CCMs are best interpreted as modular assemblies of transport, catalytic, regulatory, and structural elements that collectively shape intracellular inorganic carbon fluxes. Table 3 summarizes the key molecular components underlying microalgal CCMs, highlighting their subcellular localization, functional roles, and relative engineering potential as modular targets for rational CCM redesign.

### 3.5. Enhancement, Engineering, and Reprogramming of CCMs

**Figure 2:**
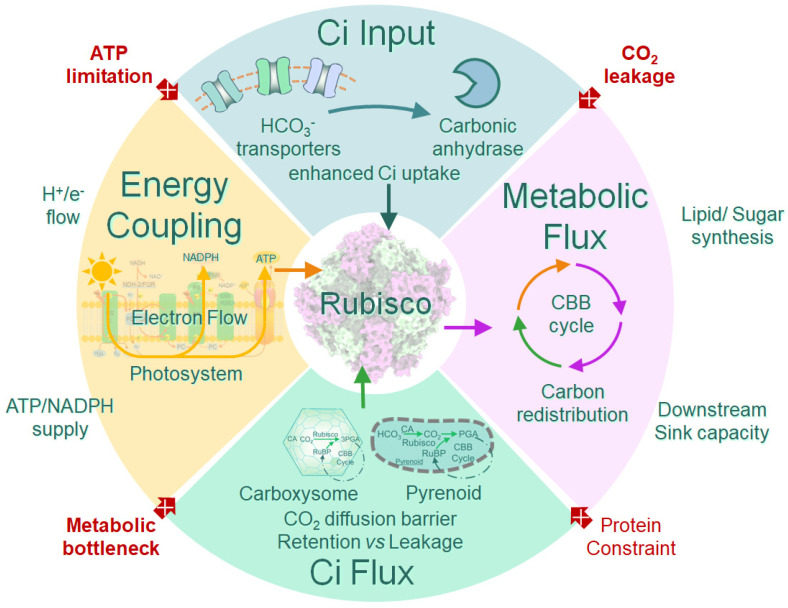
**Integrated framework for multi-scale CCM engineering in microalgae.** Core CCM mechanism operates through four interconnected modules: carbon input via HCO_3_^−^ transporters and carbonic anhydrase; energy coupling through photosystems, photosynthetic electron flow, and ATP/NADPH supply; compartmentalization via carboxysome protein shells creating CO_2_ diffusion barriers (40–80 mM local concentration); and metabolic flux distribution through CBB cycle and downstream sinks. Critical bottlenecks (red arrows) include CO_2_ leakage at compartmentalization interfaces, ATP limitation under high CCM activity, metabolic bottlenecks from insufficient downstream capacity, and protein cost constraints.

The CO_2_ concentrating mechanism constitutes a central functional module governing photosynthetic carbon fixation efficiency in microalgae. In recent years, advances in mechanistic understanding and emerging engineering efforts have shifted CCM research from descriptive characterization toward targeted manipulation. Rather than relying on single-gene interventions, recent efforts increasingly emphasize multiscale engineering strategies encompassing genetic regulation, energetic coupling, metabolic redistribution, and reactor-level integration (Figure 2). These approaches collectively hold promise for enhancing CO_2_ fixation capacity and driving the production of bio-based compounds in ways that sustain elevated carboxylation capacity under fluctuating environmental conditions [52].

#### 3.5.1. Construction of Synthetic and Enhanced CCM Module

Inorganic carbon (Ci) transporters have been identified as key regulatory nodes within CCMs. Under conditions of low CO_2_, the expression of high-affinity HCO_3_^−^/CO_2_ transporters, such as HLA3 and LCIA in green algae, or BicA and SbtA in cyanobacteria, has been associated with increased intracellular dissolved inorganic carbon (DIC) levels and improved substrate availability for Rubisco [53]. In parallel, supplementation with exogenous carbonic anhydrase (CA) or CA-associated cofactors has been applied to improve inorganic carbon availability in photobioreactors, indirectly enhancing CCM induction and operational efficiency at the system scale [54,55]. These findings imply that chromatin-state modulation could provide an additional regulatory layer for tuning CCM responsiveness [56].

Inspired by the highly efficient biophysical CCM of cyanobacteria, several studies have attempted to reconstruct key CCM components in non-native hosts, including plant chloroplasts and yeast. These efforts include partial assembly of carboxysome-like structures or heterologous expression of core structural and functional proteins (e.g., *CcmK*, *CcmL*, and *CcaA*) in plant chloroplasts or yeast, with the aim of generating localized CO_2_-enriched microenvironments [57,58]. To date, however, most reported outcomes remain at the level of proof-of-concept demonstrations or partial functional reconstruction. Achieving stable, fully operational CCMs in eukaryotic hosts remains a major challenge, constrained by issues of protein targeting, stoichiometric balance, and regulatory compatibility [33].

#### 3.5.2. Regulatory Networks and Optimization of Energy Coupling

CCM operation is energetically demanding and tightly coupled to photosynthetic electron transport, ATP synthesis, and proton-motive gradient generation [59]. Active bicarbonate transport and micro-compartmental CO_2_ conversion impose additional energetic requirements beyond baseline C_3_ photosynthesis. Consequently, enhancing photosynthetic electron flow or optimizing energy partitioning between light reactions and carbon assimilation has emerged as a promising strategy to improve CCM efficiency. Recent studies indicate that under low-CO_2_ conditions, the interactions among CCM activation, photorespiration, and mitochondrial metabolism are context-dependent. Proper coordination between these processes may alleviate metabolic bottlenecks and prevent inhibitory metabolite accumulation [49]. Dissecting such regulatory networks provides important guidance for engineering CCMs with improved robustness and stress tolerance under environmental fluctuation rather than maximizing peak performance under controlled laboratory conditions.

#### 3.5.3. System- and Metabolism-Level Reinforcement of CCMs

Insights from multi-omics analyses and genome-scale metabolic modeling underscore that enhancing CCM function requires not only increased Ci capture but also coordinated redistribution of carbon fluxes toward desired metabolic sinks. Without downstream pathway optimization, gains in carbon fixation may be offset by metabolic bottlenecks [52]. This systems-level perspective reframes CCM engineering as a flux coordination problem rather than a transporter-limited constraint. At the process level, increasing the concentration of DIC in photobioreactors by adding exogenous CA has been shown to indirectly activate CCMs, thereby improving CO_2_ uptake and photosynthetic efficiency [54]. Furthermore, coupling CCM engineering with attached growth systems, advanced reactor designs, and enhanced gas–liquid mass transfer strategies can substantially increase overall carbon capture efficiency. These system-level interventions act synergistically with intracellular CCM enhancements [60].

#### 3.5.4. Ecological Engineering and Hybrid System Integration

The objectives of CCM engineering are expanding beyond sole enhancement of photosynthetic carbon fixation toward high-value product synthesis and carbon resource utilization. One emerging approach involves co-cultivation of microalgae with selected microbial partners, enabling metabolic complementarity, improved gas exchange, and buffering against environmental fluctuations. Such strategies have demonstrated improved CO_2_ capture stability at pilot and industrial scales [60]. Within the framework of circular bioeconomy framework, integrating CCM-engineered microalgal systems with flue gas treatment, wastewater nutrient recycling, and waste valorization allows simultaneous carbon sequestration and economic value generation [61]. Recent trends further include the incorporation of real-time sensors and artificial intelligence–based control systems to dynamically optimize light intensity, aeration, and nutrient supply, thereby maintaining high CCM activity and maximizing operational efficiency [60]. Nonetheless, most of these approaches remain at laboratory or pilot scales, and their long-term industrial feasibility requires further validation.

In summary, future engineering of microalgal CCMs is transitioning from single-gene or pathway-level optimization toward integrated, cross-scale systems design. Progress will depend on resolving regulatory coupling among CCM activity, photosynthetic energy transduction, and carbon metabolism through systems modeling, while advancing synthetic CCM modules via protein engineering and data-driven design to ensure functional compatibility and robustness. Equally important is the integration of intracellular modifications with reactor-scale optimization and ecological context. Collectively, coordinated advances across molecular, metabolic, and system levels are expected to enable scalable and resilient microalgal CO_2_ capture and bioresource utilization.

## 4. Functional Diversity and Regulatory Control of Carbonic Anhydrases in Microalgae

### 4.1. Carbonic Anhydrases as Core Catalytic Nodes in CCM Function

Carbonic anhydrases (CAs) are central enzymatic components of the microalgal carbon-concentrating mechanism (CCM), catalyzing the rapid and reversible interconversion between CO_2_ and HCO_3_^−^ (CO_2_ + H_2_O ↔ HCO_3_^−^ + H^+^). Through this reaction, CAs function as critical biochemical hubs that link external inorganic carbon acquisition with CO_2_ delivery to the Rubisco carboxylation site [36,62]. CAs are ubiquitous in microalgae and other photosynthetic organisms, and their functional diversity within CCMs is determined by their phylogenetic class, subcellular localization, metal cofactor dependency, and regulatory control [63]. As such, CAs should be viewed not merely as accessory enzymes, but as dynamic regulatory hubs embedded within CCM networks.

### 4.2. Diversity and Evolution of Carbonic Anhydrase Classes in Microalgae

Microalgae possess a remarkable diversity of CA isoforms, which are distributed across multiple cellular compartments, including the chloroplast stroma, thylakoid lumen, cytosol, chloroplast membranes, and extracellular space. Collectively, these enzymes sustain elevated CO_2_ concentrations in proximity to Rubisco and thereby support efficient carbon fixation. Eight major CA classes—α, β, γ, δ, ζ, η, θ, and ι—have been identified, each differing in structural organization, phylogenetic distribution, metal dependency, and catalytic properties (Supplementary Materials Part III and Table S1). Importantly, these classes are evolutionarily unrelated despite catalyzing the same reaction, representing striking examples of convergent enzymatic evolution. Their diversification appears closely linked to ecological pressures, particularly fluctuations in CO_2_ availability and trace metal composition in aquatic environments.

CAs cannot be reliably inferred from sequence similarity alone. Subcellular targeting, metal availability, gene duplication history, and regulatory dynamics collectively shape in vivo activity [34,64,65]. Comparative genomic analyses indicate that extensive gene duplication and diversification across algal lineages, underscoring ongoing evolutionary fine-tuning driven by ecological end metabolic pressures [61].

### 4.3. Subcellular Localization and Functional Partitioning of CA Isoforms

The functional contribution of individual CA isoforms to CCMs is critically shaped by their precise subcellular localization. Distinct CA isoforms are targeted to extracellular surfaces, cytosol, chloroplast envelopes, thylakoid lumens, pyrenoids, or carboxysomes, where they perform non-redundant roles in inorganic carbon conversion and spatial CO_2_ delivery. Extracellular and periplasmic CAs facilitate the initial hydration or dehydration of CO_2_ at the cell–environment interface, thereby modulating the form of inorganic carbon available for membrane transport. Cytosolic and stromal CAs contribute to buffering intracellular HCO_3_^−^ pools and maintaining favorable chemical gradients, while lumenal or microcompartment-associated CAs generate localized CO_2_ directly adjacent to Rubisco, minimizing diffusional losses.

In complex plastid-bearing algae, including diatoms, multilayered membranes and additional intermembrane compartments impose additional spatial constraints that necessitate fine-tuned CA partitioning. CA isoforms localized to specific plastid membranes or intermembrane spaces coordinate carbon flux across barriers, effectively integrating structural compartmentalization with catalytic control. These localization patterns underscore that CA function within CCMs is inherently spatial and need to be interpreted within the architectural context of the cells [34].

### 4.4. Environmental and Cellular Regulation of CA Expression and Activity

In microalgae, CAs expression and catalytic activity are tightly regulated by multiple environmental and cellular factors, enabling dynamic adjustment of CCM performance under fluctuating environments.

CAs gene expression is highly sensitive to ambient CO_2_ levels. A rapid and pronounced upregulation of multiple CA genes has been consistently observed in a wide range of microalgal taxa, including *Chlamydomonas reinhardtii*, *Phaeodactylum tricornutum*, and *Thalassiosira* spp., under air-level CO_2_ or lower. This upregulation is mediated by CO_2_-responsive transcriptional regulators and signalling pathways [55]. In contrast, elevated CO_2_ concentrations generally suppress CA expression, although threshold responses vary among lineages [66].

Light availability strongly modulates CO_2_-dependent CA expression. Under low CO_2_ conditions, illumination enhances CA transcription, whereas darkness typically reduces expression [67]. Cellular redox status and circadian rhythms further contribute to CA regulation, with pronounced diel oscillations reported for specific CA genes [68]. Ocean acidification and reduced DIC broadly influence CA and other CCM-related genes, often leading to partial repression under high CO_2_, low-pH scenarios [69].

CA catalytic activity is critically dependent on metal cofactors. Zn^2+^ serves as the primary catalytic metal for most CA classes, and Zn limitation in marine environments frequently constrains CCM efficiency [70]. Some microalgae partially substitute Zn^2+^ with alternative metals, enabling functional resilience under trace metal limitation. Conversely, toxic heavy metals strongly inhibit CA activity, particularly extracellular CA, leading to its widespread use as a biomarker for metal pollution [71].

Enzymatic performance is additionally influenced by pH and temperature ranges, with defined optimal ranges for most isoforms [72]. Light intensity and ultraviolet radiation further modulate CA activity, with excessive irradiance causing substantial inhibition [73]. Beyond transcriptional control, post-translational modifications such as phosphorylation and redox regulation provide rapid and reversible modulation of CA activity and localization [74].

### 4.5. Engineering and Application of CAs for Enhanced Carbon Capture and Microalgal Productivity

Microalgal CO_2_ fixation represents a promising biotechnological strategy for climate change mitigation, and carbonic anhydrase has emerged as a key target for enhancing biological CO_2_ sequestration efficiency [48,72]. CA-centered applications fall into two main scenarios: improving carbon assimilation under low-CO_2_ natural environments and enhancing industrial CO_2_ capture and conversion through CCM reinforcement.

Identification and deployment of high-performance CA variants provide a foundation for such strategies [75]. Heterologous expression of thermostable and alkali-tolerant CAs, as well as supplementation with exogenous CA sources, has been shown to enhance microalgal growth, biomass productivity, and value-added product formation [76]. CA-centered microbial co-culture systems further improve overall CO_2_ fixation performance by providing complementary catalytic capacity [55].

However, effective deployment requires quantitative balance. Excessive CA expression or supplementation may dissipate intracellular inorganic carbon gradients and reduce net fixation efficiency [72]. CA therefore functions not only as a catalytic component of CCMs but also as an integrative regulatory node linking carbon uptake, energy balance, and metabolic fluxes. Its sensitivity to pH, redox state, and phosphorylation enables CA to respond dynamically to changes in light, CO_2_ availability, and cellular energy status, potentially coordinating with photosynthetic electron transport processes. Thus, CAs engineering must consider spatial organization, transporter coordination, and metabolic integration.

Future research priorities include elucidating CA–transporter–microcompartment supracomplexes [77], developing synthetic biology strategies for precise spatiotemporal control of CA activity [78], and exploring synergistic interactions between CA, photosystems, and the photosynthetic electron transport chain [79]. Such advances will strengthen the theoretical and technological foundation for scalable and resilient microalgal carbon capture platforms.

**Figure 3:**
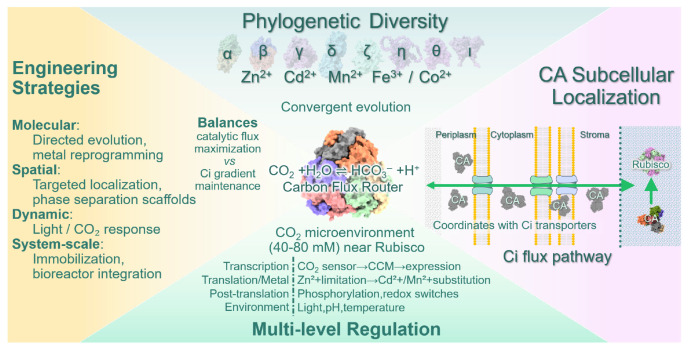
**Carbonic anhydrase (CA) as the catalytic hub and carbon flux router in microalgal carbon concentrating mechanisms (CCMs).** CAs occupy a central position within CCMs, acting as dynamic regulators that coordinate inorganic carbon (Ci) acquisition, intracellular HCO_3_^−^ homeostasis, and CO_2_ delivery to Rubisco. (**Top**) Phylogenetic diversity spans eight CA families that exemplify convergent evolution—catalyzing the same reaction despite structural non-homology—with distinct metal cofactor dependencies reflecting adaptation to trace metal availability in aquatic environments. (**Right**) Subcellular localization defines functional roles along the Ci flux pathway: extracellular/periplasmic CAs regulate interfacial CO_2_ hydration; cytosolic/stromal CAs buffer intracellular HCO_3_^−^ pools and sustain chemical gradients; thylakoid lumen and microcompartment-associated CAs generate localized CO_2_ microenvironments proximal to Rubisco within carboxysomes or pyrenoids, thereby minimizing diffusion losses. (**Bottom**) Multi-layered regulatory networks enable dynamic CCM responses, integrating transcriptional control, metal homeostasis, post-translational modification, and environmental signals. (**Left**) Engineering strategies operate across four scales: molecular optimization, spatial targeting, dynamic regulation, and system-level integration. A central design constraint is the trade-off between “catalytic flux maximization” and “Ci gradient maintenance,” as excessive CA activity can dissipate concentration gradients and reduce net carbon fixation efficiency. Collectively, this framework redefines CA from a passive catalytic component to a programmable “carbon flux router,” underpinning next-generation microalgal carbon capture and biomanufacturing systems.

CA engineering has evolved from simply enhancing catalytic rates toward multi-scale regulation of carbon flux, aiming to coordinate Ci transport, localized CO_2_ generation, and cellular energy allocation [72,80]. Within CCMs, CA is increasingly recognized as a programmable “carbon flux controller”, rather than a passive catalytic component (Figure 3).

## 5. Functions and Regulatory Control of Rubisco

### 5.1. Rubisco as the Catalytic Gatekeeper of Biological CO_2_ Fixation

Ribulose-1,5-bisphosphate carboxylase/oxygenase (Rubisco) is the only enzyme in the Calvin–Benson–Bassham (CBB) cycle that directly catalyzes biological CO_2_ fixation. It converts CO_2_ and ribulose-1,5-bisphosphate (RuBP) into two molecules of 3-phosphoglycerate (3-PGA), thereby introducing DIC into central metabolism [81]. Despite its intrinsically low carboxylation turnover rate and the competing oxygenation side reaction that initiates photorespiration, Rubisco is the most abundant protein on Earth and underpins nearly all carbon assimilation in oxygenic photosynthetic organisms [82].

Microalgae, encompassing both cyanobacteria and eukaryotic microalgae, constitute a major global carbon sink, owing to their rapid generation times (hours to days), high cell densities, and remarkably elevated volumetric Rubisco fluxes [83]. Global net primary productivity (NPP) is approximately 120 Pg C·yr^−1^, with ~50% derived from marine systems, where carbon fixation is almost entirely catalyzed by phytoplankton Rubisco [84]. Since the onset of the Phanerozoic eon, eukaryotic microalgae, predominantly red-type Rubisco-positive species, such as diatoms that have evolved through secondary endosymbiosis, have played a pivotal role in maintaining elevated levels of marine productivity [85]. For example, massive CO_2_ fixation by *Coccolithophores* during the Cretaceous profoundly reshaped Earth’s carbon cycle. Notably, satellite observations (SeaWiFS and MODIS-Aqua, 1998–2023) indicate a persistent global decline in NPP, with marine NPP decreasing by ~7.1% per decade, strongly correlated with rising sea surface temperature, enhanced upper-ocean stratification, and nutrient limitation [86]. These changes impose combined thermal and low-CO_2_ stresses, significantly constraining effective Rubisco carboxylation in microalgae.

### 5.2. Microalgal Rubisco Diversity Shaped by Endosymbiosis and CCM Coupling

Rubisco originated ~3.5–4.0 billion years ago in an anoxic, CO_2_-rich early Earth, where ancestral proteins likely functioned in non-photosynthetic metabolism [87]. Based on sequence, structure, and function, the Rubisco superfamily is classified into four major forms (Forms I–IV), reflecting adaptation to the atmospheric transition from high CO_2_/no O_2_ to low CO_2_/high O_2_ [81,87,88] (Supplementary Materials Part IV).

Microalgae preserve “living fossils” of Rubisco evolution. Green-type Rubisco (Form IB) represents an ancient cyanobacterial lineage, with moderate *S_C/O_* (≈80–101) and relatively high photorespiration; Red-type Rubisco (Form ID), optimized via secondary endosymbiosis, exhibits the highest *S_C/O_* values (≈110–238) and underpins modern marine carbon sinks [88,89]. Its oxygenation rate is only ~10–30% that of green-type Rubisco, enabling diatoms to maintain high carbon fixation under O_2_ saturated, low-CO_2_ oceanic conditions [90].

Cyanobacterial Rubisco is predominantly Form I (L_8_S_8_). Form IA (marine species such as *Prochlorococcus*, *S_C/O_* 40–60; *K_C_* 10–25 μM) is typically associated with α-carboxysomes, whereas Form IB (freshwater/terrestrial species, higher *K_cat_C*) is coupled with β-carboxysomes [91]. Rubisco occupies 80–90% of carboxysome volume and is orderly arranged via linker proteins, achieving local CO_2_ concentrations of 40–80 mM [57,58]. Recent in situ structural studies have revealed the high packing density and precise assembly of Rubisco within β-carboxysomes, providing a critical basis for CCM engineering.

Green-lineage eukaryotic microalgae (*Chlorophyta*) mainly possess Form IB Rubisco derived from primary cyanobacterial endosymbiosis, characterized by moderate or low CO_2_/O_2_ specificity and relatively low *K_cat_C* [92]. In these organisms, Rubisco interacts with the intrinsically disordered multivalent linker EPYC1 to form reversible networks that undergo liquid–liquid phase separation (LLPS), generating a Rubisco-rich pyrenoid matrix in the chloroplast stroma [93]. LLPS-driven concentration elevates local Rubisco abundance, enhances CO_2_ availability, and suppresses photorespiration, thereby sustaining high photosynthetic efficiency.

Red-lineage eukaryotic microalgae, including diatoms, brown algae, and most marine phytoplankton, predominantly use Form ID Rubisco and constitute the core of modern oceanic carbon sinks [84]. Compared with Form IB, red-type Rubisco generally exhibits higher CO_2_/O_2_ specificity and superior catalytic efficiency [90]. Meta-analyses indicate consistently elevated *S_C/O_* values in red-lineage Rubisco, with diatoms showing particularly broad kinetic diversity [94]. Consequently, diatom Rubisco, together with diverse CCM architectures, forms a highly flexible productivity strategy spectrum. Red-lineage taxa typically rely more on membrane- and organelle-level HCO_3_^−^ transporters coupled with CA to elevate CO_2_ near Rubisco, rather than strictly ordered LLPS-based pyrenoids, although mixed or partial phase-separated structures also occur depending on species and ecological niche [95].

A few dinoflagellates (e.g., *Karenia brevis*) and early-diverging eukaryotic algae retain Form II Rubisco, assembling as L_2_ dimers or higher oligomers (up to L_10_). With very low *S_C/O_*, these organisms depend on highly efficient HCO_3_^−^ uptake and pumping to maintain elevated intracellular and chloroplastic CO_2_ levels, reflecting their unique evolutionary history and ecological adaptation [96].

In summary, microalgal Rubisco function is inseparable from CCM context. Lineage-specific kinetic properties are complemented by distinct spatial concentration strategies, enabling high catalytic flux despite intrinsic limitations such as low turnover and unavoidable oxygenation. This tight coupling between enzyme evolution and carbon-concentrating architecture underpins the ecological success of microalgae across diverse aquatic environments.

### 5.3. Regulation of Rubisco Expression and Catalytic Activity in Microalgae

Rubisco is the central rate-limiting enzyme of carbon assimilation in photosynthetic microalgae. Its cellular abundance and effective catalytic capacity are highly plastic and subject to multilayered regulation by environmental cues and cellular metabolic status. Overall, microalgae balance carbon fixation efficiency and resource allocation through demand-driven control of Rubisco expression and activity maintenance under fluctuating conditions. Rubisco biosynthesis (encoded by *rbcL*/*rbcS*) is primarily coordinated by inorganic carbon availability, light conditions, and nitrogen status, while integrating circadian rhythms, temperature, and multiple abiotic stress signals. Regulation operates at transcriptional, post-transcriptional, translational, and protein homeostasis levels, among which transcriptional activation and selective protein degradation are the dominant determinants of Rubisco abundance.

Inorganic carbon availability represents the strongest regulatory signal for Rubisco expression [56]. Under CO_2_/HCO_3_^−^ limitation, microalgae rapidly activate carbon-concentrating mechanisms (CCMs), accompanied by pronounced upregulation of *rbcL*/*rbcS* transcription. In contrast, elevated external CO_2_ generally suppresses Rubisco expression [97]. This response is mediated by CCM-associated transcriptional regulators, such as CCM1/CIA5 in green algae and CmpR or CRP in cyanobacteria, enabling dynamic coordination between Rubisco synthesis and CO_2_ supply capacity [98].

Light intensity and spectral quality regulate Rubisco expression indirectly by modulating carbon demand. Rubisco genes typically exhibit strong light inducibility, under the control of red- and blue-light sensing systems [99]. High irradiance enhances photosynthetic electron transport and carbon fixation demand, leading to increased Rubisco expression [100]. Conversely, in darkness or prolonged low-light conditions, Rubisco, particularly in green algae, is degraded rapidly, reflecting the active recovery of nitrogen and energy resources [101].

As one of the largest nitrogen reservoirs in photosynthetic cells, Rubisco synthesis and turnover are highly sensitive to nitrogen supply [102]. Nitrogen availability exerts central control over carbon fixation pathways and associated components. Because Rubisco can constitute a substantial fraction of total cellular protein in many species [103], nitrogen limitation leads to marked reductions in Rubisco content, whereas nitrogen resupply rapidly restores expression and holoenzyme assembly in a reversible manner [104]. Different inorganic nitrogen forms (e.g., nitrate versus ammonium) also function as signaling molecules, directly influencing *rbcL*/*rbcS* transcription [105].

Transcriptomic and proteomic studies in multiple microalgae and cyanobacteria reveal pronounced diel oscillations in Rubisco abundance and its associated network, typically peaking during the light phase and declining at night [106]. This rhythmic regulation reflects coordination between endogenous circadian clocks and light–dark cycles to optimize photosynthetic resource allocation [107]. Growth temperature further influences the composition and relative abundance of Rubisco small subunit isoforms and is often accompanied by changes in enzyme kinetics, indicating that temperature modulates not only expression but also assembly state and functional properties [108].

Various abiotic stresses also affect Rubisco expression and activity. Under salinity stress, oxidative stress, or nutrient limitations (e.g., phosphorus or iron), the abundance of Rubisco and Rubisco activase (RCA) typically declines, resulting in reduced carbon fixation capacity [109]. These responses likely reflect physiological trade-offs associated with energy allocation, redox balance, and osmotic regulation. In particular, oxidative stress and P or Fe limitation suppress Rubisco expression to reduce nitrogen and energy expenditure [110]. In summary, regulation of Rubisco expression in microalgae reflects highly integrated sensing and dynamic allocation of carbon, nitrogen, and energy resources, forming a fundamental basis for growth and survival in complex and fluctuating environments [107].

In vivo Rubisco carboxylation efficiency depends not only on enzyme abundance but also on substrate availability (CCM performance), maintenance of activation state via RCA, and environmental temperature. Together, these factors determine the effective catalytic flux and operational stability of Rubisco. Proper structure and assembly are prerequisites for catalytic function. Canonical Form I Rubisco assembles as an L_8_S_8_ hexadecamer, in which the large subunit (RbcL) contains the active site, while the small subunit (RbcS), although non-catalytic, modulates conformational dynamics, substrate specificity, and complex stability [111]. Rubisco folding and assembly require multiple chaperone systems, including *RbcX*, *Raf1*, and *GroEL–GroES*/*Cpn60*, whose expression ratios and assembly timing critically influence final catalytic performance [112]. Reports of non-canonical assemblies (e.g., L_8_S_4_) in some algae suggest that Rubisco structural diversity remains incompletely understood.

The in vivo activity of Rubisco is commonly expressed as the ratio of initial to total activity, reflecting the proportion of catalytically competent enzyme. The core regulatory step is carbamylation of a key lysine residue by non-substrate CO_2_, followed by Mg^2+^ coordination, as well as efficient removal of inhibitory sugar phosphates [113]. Rubisco activase (RCA) maintains enzyme availability by ATP-driven conformational remodeling that removes inhibitors such as RuBP and CA1P. The type and thermal stability of RCA largely determine the temperature response of Rubisco. Microalgal RCA exhibits high diversity. For example, *Chlamydomonas reinhardtii* possesses both a heat-sensitive α-type RCA and a relatively thermostable β-type, whereas diatoms and red algae generally harbor more thermotolerant RCAs with optimal activity around 40–45 °C [114]. Thermostable RCA variants identified in certain species provide valuable templates for improving Rubisco performance under warming conditions [115].

Substrate availability is a primary determinant of actual carboxylation rate [116]. Under fully functional CCMs, CO_2_ microenvironments within pyrenoids (microalgae) or carboxysomes (cyanobacteria) greatly exceed ambient levels, allowing Rubisco to operate near substrate saturation and sustain high catalytic fluxes [95]. Conversely, CCM impairment—caused by high temperature, acidification, or nutrient limitation—rapidly reduces local CO_2_ concentrations, leading to pronounced declines in Rubisco activity and carbon fixation rates [56]. Finally, CO_2_/O_2_ ratios, pH, and diel regulation exert fine control over Rubisco activity [113]. Low CO_2_ or elevated O_2_ favors the oxygenation reaction and enhances photorespiration. Rubisco typically exhibits maximal activity under mildly alkaline conditions (pH ≈ 7.8–8.3) [117]. In some green algae, nocturnally synthesized CA1P transiently inhibits Rubisco and is removed at dawn via light-dependent RCA activity; however, the prevalence and significance of this mechanism vary widely among microalgal taxa [117].

### 5.4. Engineering Strategies for Rubisco-Mediated Carbon Fixation in Microalgae

**Figure 4:**
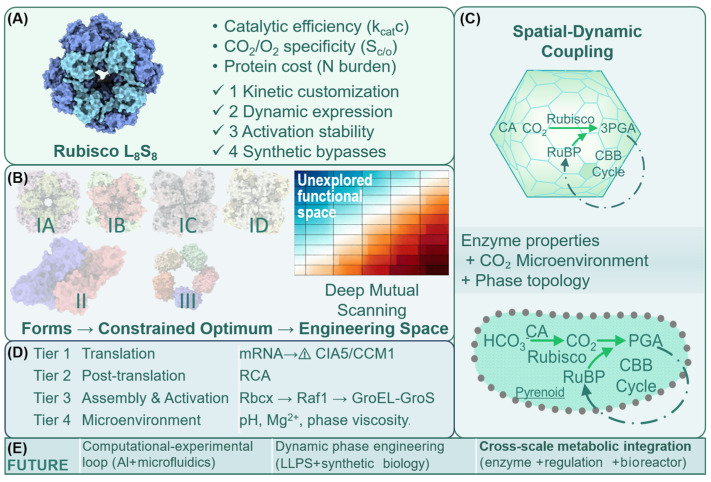
**Rubisco as a systemic constraint node and engineering target in microalgal carbon fixation.** (**A**) Rubisco defines the entry point of the CBB cycle and acts as a constraint node where catalytic efficiency, CO_2_/O_2_ specificity, and nitrogen cost converge to limit carbon flux. (**B**) Evolutionary diversification reveals different Rubisco form I, form II and form III, form I is further subdivided into IA (marine cyanobacteria), IB (green algae and land plants), IC (some bacteria), and ID (red algal lineage); recent studies redefine it as possessing substantial unexplored engineering potential. (**C**) Spatial–dynamic coupling: carboxysomes and pyrenoids generate localized CO_2_ microenvironments that determine in vivo enzyme performance. (**D**) Multi-level regulation integrates transcriptional control, assembly, activation, and microenvironmental constraints. (**E**) Future engineering converges on three directions: AI-guided design and high-throughput screening, phase-based spatial engineering, and cross-scale metabolic integration.

Actually, Rubisco’s inherently low carboxylation efficiency, high nitrogen demand, and sensitivity to environmental fluctuations remain major bottlenecks for large-scale applications [89]. In practical cultivation, light, temperature, and CO_2_ concentration are highly dynamic; merely improving intrinsic Rubisco kinetics is insufficient to meet the combined demands of resource-use efficiency, environmental robustness, and system scalability [89]. Consequently, Rubisco engineering has shifted from single-enzyme optimization toward a multi-scale, application-driven framework encompassing enzyme kinetics, expression regulation, activation maintenance, spatial organization, and integration with alternative carbon-fixation pathways [118]. The following sections summarize representative engineering strategies (Figure 4).

Natural Rubisco exhibits remarkable catalytic diversity, with different Form I enzymes showing trade-offs between carboxylation specificity (*S_C/O_*) and turnover rate (*K_cat_C*). Red algal and some diatom Rubiscos generally possess high CO_2_ specificity, while thermophilic red algae and certain chemoautotrophs exhibit higher *K_cat_*, providing templates to overcome traditional kinetic constraints [85]. The “optimal Rubisco” is context-dependent, requiring tailored selection based on local CO_2_ microenvironments [119]. Under high CO_2_ or concentrated conditions, introducing high-*K_cat_* Rubisco is a viable strategy to enhance per-unit-time carboxylation flux. Advances in chloroplast genome editing, heterologous expression, and chaperone-assisted assembly have enabled functional verification of Rubisco in foreign hosts [120]. Structural biology and high-throughput mutagenesis continue to reveal the roles of active sites, Loop 6, and small-subunit interfaces in modulating substrate specificity and conformational dynamics, providing a molecular basis for context-adapted Rubisco design [88].

Rubisco represents a major nitrogen reservoir in photosynthetic cells, and constitutive high-level expression under nitrogen limitation can reduce overall photosynthetic efficiency and nitrogen-use efficiency [121]. Therefore, engineering efforts are shifting from absolute carboxylation capacity toward maximizing carbon fixed per unit nitrogen (photosynthetic nitrogen-use efficiency, PNUE) [121]. In microalgae, tightly controllable culture conditions allow the use of light- or CO_2_-responsive regulatory elements to match Rubisco abundance to instantaneous carbon demand. This strategy preserves photosynthetic capacity while substantially reducing nitrogen consumption, offering practical benefits for high-density cultivation and potential guidance for nitrogen-efficient crop engineering.

In vivo Rubisco activity critically depends on Rubisco activase (RCA), which removes inhibitory sugar phosphates (e.g., RuBP, CA1P) via ATP-driven conformational remodeling [113]. RCA thermal stability often limits photosynthetic efficiency under stress conditions. Consequently, engineering strategies now emphasize system-level optimization linking Rubisco, RCA, energy supply, and spatial organization [122]. Screening or engineering thermotolerant RCAs to maintain activation at elevated temperatures has been emphasized [123]. Enhancing energy matching between light reactions and carbon assimilation at the metabolic network level can reduce the impact of RCA limitations on carboxylation flux [124]; mitigating oxygenation reactions and photorespiration can also buffer RCA constraints. Spatial organization, such as pyrenoid formation or synthetic microcompartments, can locally elevate CO_2_ around Rubisco without altering the enzyme itself [125]. Pyrenoids facilitate high-density Rubisco aggregation and coordinate with carbonic anhydrase, significantly enhancing substrate supply [126]. These strategies bridge molecular performance with system-level photosynthetic efficiency, particularly valuable for high-density microalgal cultivation and thermally challenging environments.

Synthetic biology has introduced non-natural or semi-artificial carbon-fixation pathways to bypass Rubisco’s kinetic and thermodynamic limitations, such as the Crotonyl-CoA/ethylmalonyl-CoA/hydroxybutyryl-CoA cycle (CETCH cycle) and C_1_ assimilation strategies. The CETCH cycle demonstrates theoretically higher carbon efficiency and enzyme flux than the Calvin–Benson–Bassham (CBB) cycle, avoiding oxygenation reactions and improving energy retention [127]. CO_2_ can first be converted into reactive intermediates (e.g., formate or formaldehyde) and then assimilated into central metabolism, partially circumventing Rubisco’s rate limitation [128]. Functional expression has been demonstrated in heterotrophic or mixotrophic systems, with promising energy-to-carbon conversion efficiency. Current efforts focus on system-level integration with CBB cycles to offload Rubisco flux by acting as supplemental carbon sinks [127], dynamically activate alternative pathways under stress (high light, temperature, or low CO_2_), and combine with CCMs or synthetic microcompartments to reduce metabolic conflicts [129]. Most non-natural pathways remain at the proof-of-concept stage in vitro or in heterotrophic models. Their long-term stability, metabolic compatibility, regulatory complexity, and ecological safety in photosynthetic hosts remain major challenges [130]. In the near term, these pathways are better viewed as supplementary insurance mechanisms rather than replacements for the CBB cycle. Nevertheless, they offer a design space for next-generation artificial carbon-fixation systems: Rubisco serves as the central engine, surrounded by tunable auxiliary modules enabling dynamic flux partitioning, spatial organization, and metabolic coupling, achieving high throughput, robustness, and resource efficiency. Microalgae, with their genetic tractability, photosynthetic plasticity, and scalable cultivation potential, are ideal platforms for testing and iterating such complex carbon-fixation networks.

## 6. Perspectives and Future Directions

Despite advances in enhancing Rubisco kinetics and reactor hydrodynamics, several fundamental constraints continue to limit the thermodynamic and economic efficiency of microalgal CO_2_ fixation. A primary challenge is the spatiotemporal mismatch between photon flux input, which occurs on a millisecond timescale, and the significantly slower rates of carboxylation and metabolic turnover, leading to inevitable energy dissipation and photoinhibition. Furthermore, the non-linear coupling between CO_2_ supply, CCM gene expression, and metabolic flux redistribution is not yet fully quantified, making it difficult to predict system behavior under fluctuating conditions. In large-scale reactors, multiphysics heterogeneity presents another obstacle, where uneven distributions of light, flow fields, and temperature create inefficient dead zones that limit precise process control. Industrial stressors also pose a stability challenge, as the long-term physiological impact of impurities in industrial flue gas, such as sulfur and nitrogen oxides or heavy metals, remains poorly understood. Overcoming these limitations requires a shift from single-factor optimization to multiscale systemic integration, linking molecular design directly to macroscopic reactor performance.

To address these biological limitations, the integration of systems biology and synthetic biology offers tools to transform microalgae from evolved organisms into purpose-built carbon-fixing chassis. Moving beyond native mechanisms, future efforts should focus on the rational design of artificial CCMs, including the heterologous assembly of efficient C_4_ enzymatic systems like PEPC and PPDK, or the introduction of non-native pathways such as the CETCH cycle or acetyl-CoA bypasses to decouple carbon fixation from strict photorespiratory constraints. Complementing this is the application of precision gene editing and multigene tuning via CRISPR-Cas technologies, which allows for the simultaneous regulation of multiple targets. Engineering strategies must evolve from single-gene overexpression to the coordinated tuning of entire networks, synchronizing CCM components, Rubisco activation systems, and lipid biosynthesis pathways to eliminate metabolic bottlenecks. As genetically engineered strains move towards outdoor deployment, ecological risk assessment becomes paramount. The development of genetic firewalls, such as auxotrophy dependencies or programmable self-destruct circuits, will be essential to prevent environmental escape and ensure regulatory compliance and public acceptance.

Concurrent with biological rewiring, the future of cultivation lies in the transition from static operation to adaptive, intelligence-driven control systems, often termed the Intelligent Photosynthetic Factory. A major frontier is the development of digital twins and multiscale modeling, which couple Computational Fluid Dynamics (CFD) with genome-scale metabolic models (GEMs) and light transmission kinetics. These holistic models will enable predictive optimization of gas–liquid mass transfer and metabolic responses, guiding reactor design in silico before physical prototyping. Furthermore, integrating Internet of Things (IoT) sensors with machine learning algorithms enables AI-enabled feedback loops capable of real-time control. Future systems will dynamically adjust light intensity, spectral quality via programmable LEDs, and CO_2_ dosing in response to instantaneous physiological states, maximizing light-use efficiency. Advanced control logic must also move beyond maintaining constant setpoints to implementing light–temperature–carbon synergistic control, utilizing dynamic regimes such as pulsed light or temperature cycling to exploit the natural rhythmic properties of algal metabolism for enhanced productivity.

Finally, to achieve economic viability and carbon neutrality, microalgal systems must be integrated into broader industrial and ecological cycles through circular bioeconomy principles and process intensification. The waste-to-resource integration strategy, coupling CO_2_ fixation with wastewater treatment for nutrient recovery and industrial exhaust purification, represents the most viable path for cost reduction. Developing extremophilic strains capable of directly utilizing raw flue gas and degrading NOx/SOx is a priority. However, economic models dictate that carbon sequestration alone is rarely profitable; thus, the biorefinery approach must prioritize the co-production of high-value compounds alongside biofuels. Rigorous Life Cycle Assessment (LCA) and Techno-Economic Analysis (TEA) must guide technology selection, ensuring that the energy inputs for construction and operation do not negate the carbon sequestered, thereby truly realizing the goal of negative-emission technologies. In conclusion, the future of microalgal CO_2_ fixation lies in the end-to-end integration of molecular synthetic biology, intelligent process engineering, and circular economic principles. By resolving the bottlenecks of energy efficiency and scalability, microalgae are poised to become a cornerstone technology in the global transition toward a sustainable, carbon-neutral society.

## 7. Conclusions

Microalgal CO_2_ fixation is a multiscale process governed by gas–liquid mass transfer, photosynthetic energy conversion, CCM activity, enzymatic regulation, metabolic flux allocation, and environmental control. This review shows that CO_2_ delivery from the gas phase to the culture medium remains a major upstream bottleneck, while efficient intracellular fixation depends on coordinated CCM–carbonic anhydrase–Rubisco function rather than isolated optimization of single components. The evidence further indicates that future scale-up will rely on system-level integration linking biological engineering, environmental regulation, and intelligent process control. Overall, the field is moving from single-factor improvement toward integrated design spanning molecular mechanisms to reactor operation. Continued advances in synthetic biology, artificial intelligence, systems modeling, and green engineering are expected to improve efficiency, robustness, and scalability, strengthening the role of microalgal CO_2_ fixation in carbon-neutral and sustainable low-carbon development.

## Figures and Tables

**Table 1:** Comparison of conventional microalgal cultivation systems in terms of CO_2_ gas–liquid mass transfer.

Reactor Type/Configuration	*K_L,a_* (s^−1^)	CO_2_ Utilization Efficiency (%)	Technology Maturity	Suitable Microalgae Species	Reference
Open raceway pond	0.0014–0.0042	20–30	Commercialisation	*Spirulina*, *Chlorella*, *Dunaliella salina*	[11]
Bubble column PBR	0.0055–0.0111	40–60	Laboratory-Pilot Scale	*Chlamydomonas*, *Microcystis*	[10]
Airlift PBR	0.0111–0.0222	50–70	Pilot Scale-Commercialisation	*Chlorella*, *Dunaliella salina*	[12]
Flat-panel PBR	0.0083–0.0167	45–65	Pilot Scale	*Scenedesmus*, *Chlorella*	[13,14]
Micro-Nano Bubble PBR	0.0222–0.0417	60–80	Laboratory Stage	Suitable for a variety of microalgae species	[15]

**Table 2:** Functional classification and mechanistic features of carbon concentrating mechanisms in microalgae.

Type	Core Mechanism	Representative Species	Key Features	References
Biophysical CCM	Active uptake of HCO_3_^−^ combined with strategic localization of carbonic anhydrases to create a high CO_2_ microenvironment around Rubisco	Cyanobacteria, green algae (e.g., *Chlamydomonas reinhardtii*), diatoms	Most common; relies on membrane transporters and CA localization to enhance substrate availability for Rubisco	[33]
Biochemical CCM/C_4_-like	Enzyme-mediated conversion of CO_2_ into C_4_ dicarboxylic acids via PEPC, MDH, and ME, followed by biochemical CO_2_ transport between intracellular compartments	Diatoms (*Thalassiosira weissflogii*, *Phaeodactylum tricornutum*), some green algae	Analogous to C_4_ plants, but completed within a single cell; hence referred to as “C_4_-like”	[34]
Hybrid CCM	Combines HCO_3_^−^ active uptake with C_4_-like cycle, using multi-layered regulation to optimize CO_2_ supply	Certain diatoms, dinoflagellates, golden algae	Mechanism can switch depending on carbon source and light intensity, providing adaptive flexibility	[3]

**Table 3:** Modular components of microalgal carbon concentrating mechanisms and their engineering relevance.

	Gene/Protein	Subcellular Localization	Functional Role	Engineering Potential
CO_2_ transport system	RHP1, CCP1/2	Plasma membrane	Facilitates direct CO_2_ uptake	Medium
HCO_3_^−^ transport system	HLA3, SULTR2	Plasma membrane	High-affinity HCO_3_^−^ transport	High
Chloroplast HCO_3_^−^ transporters	LCIA, BEST1	Chloroplast membrane	Mediates HCO_3_^−^ import into chloroplast	High
Carbonic anhydrases (CAs)	CAH1–9	Extracellular/Cytosol/Chloroplast	Catalyze reversible CO_2_ ⇌ HCO_3_^−^ conversion	Very high
Regulatory factors	Cia5, CcmR	Nucleus	Global transcriptional regulation of CCM genes	High
Protein scaffold components	EPYC1, CBC1/2	Chloroplast	Promote Rubisco aggregation and local CO_2_ concentration	Medium

## Data Availability

All data associated with the paper are included in the main document and Supplementary Materials.

## Supplementary Materials

The following supporting information can be downloaded at: https://www.mdpi.com/article/10.3390/microorganisms14050999/s1, Figure S1: Bibliometric analysis of microalgal carbon fixation research; Table S1: Basic characteristics of various types of CA related to microalgae. References [131,132,133,134,135,136,137,138,139,140,141,142,143,144,145,146,147,148] are cited in the Supplementary Materials.
