# Supplementary material for: Engineering and Biological Mechanisms of Microalgal CO2 Fixation: A Review from Molecular Regulation to System Optimization
Source: Microorganisms. 2026 Apr 29;14(5):999. doi: 10.3390/microorganisms14050999 (PMC13209882; doi:10.3390/microorganisms14050999)
Supplement: Supplementary file 1 [file microorganisms-14-00999-s001.zip › microorganisms-4241140-supplementary.pdf]

Supplement material:

Review

Engineering and Biological Mechanisms of Microalgal CO_2 Fixation: A Review from Molecular Regulation to System Optimization

Zhongliang Sun [1,*,†], Weixian Chen [2,†], Yu Xie [1], Shoukai Guo [1], Liqin Sun [1] and Qiang Wang [3,*]

[1] School of Life Sciences, Yantai University, Yantai 264005, China; 202500370042@s.ytu.edu.cn (Y.X.); 1831304192@s.ytu.edu.cn (S.G.); sliqin2005@ytu.edu.cn (L.S.)
[2] Institute of Systems, Molecular and Integrative Biology, University of Liverpool, Liverpool L69 7ZX, UK; wxchen@liverpool.ac.uk
[3] State Key Laboratory of Crop Stress Adaptation and Improvement, School of Life Sciences,
Henan University, Kaifeng 475004, China
* Correspondence: zlsun@ytu.edu.cn (Z.S.), wangqiang@henu.edu.cn (Q.W.)
† These authors contributed equally to this work.

Table of content of the Supplement material

Part I. Literature Retrieval Strategy and Bibliometric Analysis *(page 3-8)*

1.1 Blue cluster: coupling microalgal growth, carbon fixation, and bioenergy production *(page 4)*

1.2 Yellow cluster: CO_2 conversion and biocatalytic mechanisms *(page 4)*

1.3 Green cluster: photosynthesis and carbon concentrating mechanisms (CCMs) *(page 5-6)*

1.4 Red cluster: ecosystem-level and natural carbon sequestration processes *(page 6)*

1.5 Integrated perspective and structure of this review *(page 6-8)*

Part II. Functional types and conceptual frameworks of Biophysical CCMs *(page 9-12)*

2.1 Carboxysome-Based CCMs *(page 9)*

2.2 Pyrenoid-Based CCMs *(page 9-11)*

2.3 Biophysical CCMs in *Coccolithophores (page 11-12)*

Part III. Diversity and evolution of carbonic anhydrase classes in microalgae *(page 13-15)*

Part IV. Evolutionary origin and major forms of the Rubisco superfamily (Forms I–IV) *(page16-17)*

Part V. Reference *(page18-21)*

1. Literature Retrieval Strategy and Bibliometric Analysis

To systematically characterize the knowledge structure and evolving research frontiers in microalgal carbon fixation, bibliometric data were retrieved from the Web of Science Core Collection using the following query:

TS = (*microalgae* OR *microalga* OR "*micro alg*" OR *cyanobacteria* OR *algae*) AND TS = ("*carbon concentrating*" OR "*carbon fixation*" OR "*CO_2 fixation*" OR "*carbon sequestration*" OR "*CO_2 capture*").

The retrieved records were analyzed using VOSviewer (v1.6.20) to construct a keyword co-occurrence network and knowledge map of the field (***Figure S1***).

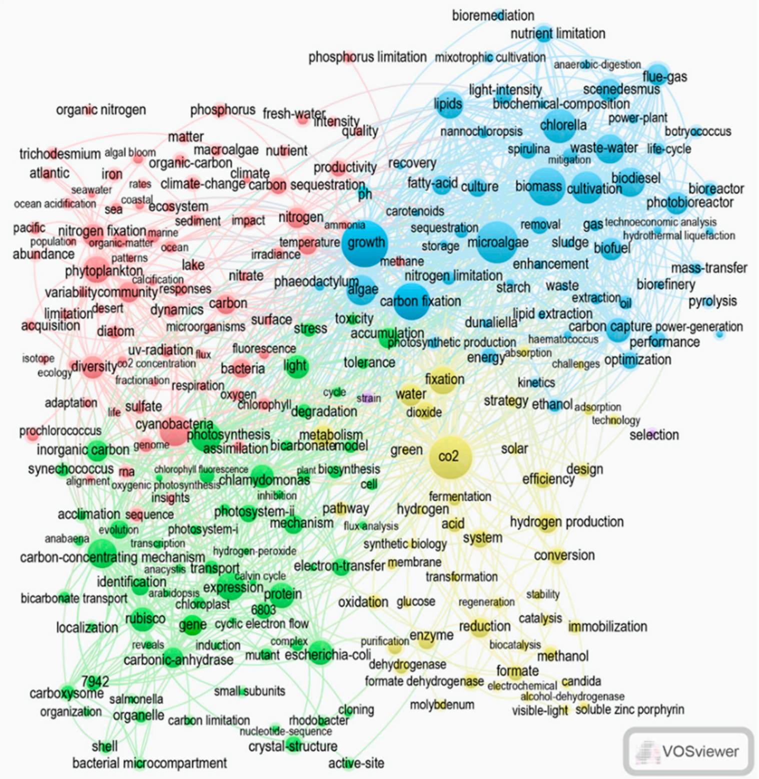

***Figure S1** Bibliometric analysis of microalgal carbon fixation research*

The resulting network exhibits a highly modular yet interconnected structure. High-frequency keywords such as growth, microalgae, CO_2, photosynthesis, and phytoplankton form the core of the network, indicating that growth regulation-

photosynthesis-carbon fixation-ecosystem processes constitute the long-standing research backbone of microalgal CO_2 fixation. Based on clustering analysis, the keyword network can be divided into four major functional modules, corresponding to distinct research scales and technological pathways.

1.1 Blue cluster: coupling microalgal growth, carbon fixation, and bioenergy production

The blue cluster is centered on growth, carbon fixation, microalgae, biomass, and cultivation, reflecting application-oriented research that integrates microalgal carbon sequestration with environmental remediation and resource recovery. The frequent co-occurrence of wastewater, flue gas, power plant, and bioremediation highlights extensive exploration of microalgae for industrial CO_2 capture and multi-pollutant wastewater treatment, aligning closely with emerging concepts of negative emission biotechnologies and carbon capture and utilization (CCU).

This cluster is strongly coupled with energy-related keywords such as lipids, biodiesel, biofuel, methane, and hydrogen, indicating a clear shift from improving carbon fixation efficiency alone toward integrated "carbon fixation-energy production" systems. Notably, the strong associations between lipids/fatty acids and light intensity or nutrient/nitrogen limitation underscore cultivation-based strategies for redirecting carbon flux toward high-value products. In parallel, keywords such as (photo)bioreactor, hydrodynamics, and mixotrophic emphasize the importance of reactor design and mass transfer enhancement, while the emergence of techno-economic analysis (TEA) signals increasing attention to process scalability and industrial feasibility.

1.2 Yellow cluster: CO_2 conversion and biocatalytic mechanisms

The yellow cluster is organized around CO_2 as the central node and is closely associated with photosynthesis, carbon concentrating mechanism, cyanobacteria, and microalgae. High-frequency keywords within this cluster include reduction, enzyme, hydrogen production, formate, methanol, and metabolism. This module reflects a paradigm shift from maximizing CO_2 fixation rates to directing fixed carbon toward targeted chemical and energy carriers.

Recent advances in utilizing photosynthetic microorganisms or synthetic biology platforms to convert CO_2 into formate, methanol, and hydrogen highlight the growing integration of carbon fixation with energy systems. The appearance of heterotrophic model organisms such as *Escherichia coli* further indicates an expanding research focus on cross-species metabolic coupling and modular CO_2 reduction pathways, extending microalgal carbon fixation beyond native photosynthetic hosts.

1.3 Green cluster: photosynthesis and carbon concentrating mechanisms (CCMs)

The green cluster, dominated by photosynthesis and carbon concentrating mechanism (CCM), represents the most fundamental yet technologically promising research direction in microalgal carbon fixation. Core keywords include light, electron transfer/flow, bicarbonate transport, Rubisco, carbonic anhydrase, and carboxysome, with model systems primarily involving *Chlamydomonas reinhardtii* and cyanobacteria such as *Synechocystis* and *Synechococcus*.

This cluster delineates the complete mechanistic framework by which CO_2 is transported across membranes, intracellularly concentrated, and delivered at elevated

local concentrations to Rubisco catalytic sites. These insights provide a theoretical foundation for CCM engineering, Rubisco performance optimization, and artificial photosynthesis design. The strong co-occurrence of light with CO_2, growth, and biomass further highlights light regulation as a critical bridge linking molecular mechanisms to applied performance optimization.

1.4 Red cluster: ecosystem-level and natural carbon sequestration processes

The red cluster, centered on cyanobacteria and phytoplankton, primarily reflects studies of community structure and environmental responses of microalgal carbon fixation in natural ecosystems. Keywords such as community, diversity, nitrogen fixation, and phosphorus, together with key functional groups including diatoms, *Prochlorococcus*, and *Trichodesmium*, emphasize the role of microalgae in global carbon-nitrogen-phosphorus biogeochemical cycles.

The frequent occurrence of climate change, temperature, ocean acidification, and algal bloom indicates a growing focus on the responses and feedbacks of natural carbon sequestration under global change scenarios. Meanwhile, the clustering of multi-omics-related keywords reflects the increasing application of molecular ecological approaches to unravel carbon fixation mechanisms in natural communities, with a predominant emphasis on marine ecosystems.

1.5 Integrated perspective and structure of this review

The bibliometric clustering analysis reveals a coordinated, multiscale evolution of microalgal CO_2 fixation research spanning molecular mechanisms, engineering applications, carbon conversion strategies, and ecosystem-level processes. Collectively,

these clusters reflect a progressive shift from descriptive physiological studies toward increasingly system-oriented and engineering-driven approaches. However, the relative density and connectivity of keywords associated with carbon concentrating mechanisms, intracellular inorganic carbon transport, Rubisco catalysis, and photosynthetic regulation highlight that mechanistic understanding of CO_2 delivery and fixation within microalgal cells remains the central axis of the field.

Guided by these insights, the present review is structured along the continuum of CO_2 supply, intracellular concentration, enzymatic fixation, and regulatory optimization. Specifically, we focus on engineering constraints and intensification strategies governing gas–liquid CO_2 mass transfer, followed by a detailed synthesis of carbon concentrating mechanisms and their functional coordination with carbonic anhydrases and Rubisco. Emphasis is further placed on metabolic regulation and environmental control as critical interfaces linking molecular mechanisms to cultivation performance. While ecosystem-scale carbon sequestration and non-photosynthetic CO_2 reduction pathways constitute important and rapidly growing research directions, they are discussed only insofar as they inform mechanistic understanding or system-level design of microalgal CO_2 fixation processes.

By explicitly integrating bibliometric evidence with mechanistic and engineering perspectives, this review aims to provide a coherent framework that bridges molecular regulation and reactor-scale optimization. This structure not only reflects the dominant knowledge architecture of the field but also facilitates the identification of key regulatory and design nodes for future innovation in efficient, intelligent, and scalable

microalgal CO_2 fixation systems.

2. Functional types and conceptual frameworks of Biophysical CCMs

2.1 Carboxysome-Based CCMs

Carboxysomes are proteinaceous bacterial micro-compartments that co-encapsulate Rubisco and carbonic anhydrase, representing the central CO_2-concentrating structure in cyanobacteria (Kerfeld and Melnicki 2016). Plasma membrane HCO_3^- transporters, such as *BicA*, *SbtA*, and *BCT1*, actively import inorganic carbon into the cytosol, establishing a high intracellular Ci pool. Within the carboxysome, CA rapidly converts accumulated HCO_3^- into CO_2, which is immediately fixed by nearby Rubisco (Huffine, et al. 2023).

The protein shell of the carboxysome imposes a diffusion barrier to CO_2, thereby minimizing leakage and amplifying CO_2 concentrations around Rubisco. This shell is composed of multiple paralogous proteins (e.g., *CcmK, CcmL, CcmN*), whose pore sizes and permeability properties govern small-molecule fluxes and influence internal pH and CO_2/ HCO_3^- equilibria. Recent experimental and modeling studies demonstrate that shell permeability and internal chemical microenvironments jointly determine carboxysome efficiency (Trettel, et al. 2024).

2.2 Pyrenoid-Based CCMs

In eukaryotic microalgae, CO_2 concentration predominantly relies on the pyrenoid, a Rubisco-rich micro-compartment located within the chloroplast. Pyrenoid assembly is generally driven by liquid–liquid phase separation (LLPS) and exhibits dynamic structural remodeling in response to ambient CO_2 conditions (Meyer, et al. 2020).

The green alga *Chlamydomonas reinhardtii* represents the most intensively characterized pyrenoid-based CCM. Its pyrenoid comprises a Rubisco-dense matrix, traversing thylakoid tubules, and an enveloping starch sheath. Multivalent interactions between Rubisco and linker proteins – most notably EPYC1 (Essential Pyrenoid Component 1) – have been shown to drive phase separation and structural organisation of the pyrenoid. These architectural and functional features have been validated through combined structural and cell biological approaches (Meyer, et al. 2020). Green algal CCMs depend on coordinated activity of HCO_3^- transporters in chloroplast membranes, extracellular or membrane-associated carbonic anhydrases, and CA isoforms localized to distinct chloroplast subcompartments. Together, these elements precisely control the spatial and temporal conversion of HCO_3^- to CO_2. Under low- CO_2 conditions, induction of HCO_3^- transport and CA expression is particularly pronounced, and Rubisco aggregation within the pyrenoid is essential for CCM functionality (He, et al. 2023).

In marine diatoms, pyrenoid-like Rubisco enrichment zones are often associated with distinct protein or membrane architectures that differ from those of green algae, reflecting adaptations to marine environments (Pierella Karlusich, et al. 2021). Genomic and functional studies reveal that most diatoms encode SLC4-family HCO_3^- transporters, which directly import bicarbonate from seawater and constitute a key molecular basis for DIC accumulation and localized CO_2 generation (Shen, et al. 2017).

Although carboxysomes and pyrenoids represent divergent evolutionary solutions in prokaryotes and eukaryotes, respectively, both achieve the same functional goal:

enhancing Rubisco carboxylation through localized CO_2 enrichment (Meyer, et al. 2020). Distinct transporter repertoires and CA complements across lineages underlie differential CCM efficiencies and environmental responses, while advances in cryo-electron tomography and phase separation reconstitution are rapidly refining mechanistic understanding and identifying engineering targets (He, et al. 2023).

2.3 Biophysical CCMs in *Coccolithophores*

Coccolithophores, typified by *Emiliania huxleyi*, have evolved a distinctive form of biophysical CCM that must simultaneously support photosynthetic CO_2 fixation and intracellular calcification. In addition to maintaining high CO_2 levels around Rubisco, coccolithophores continuously supply DIC to specialized coccolith vesicles, where calcium carbonate precipitation occurs. Consequently, DIC uptake, allocation, and redistribution are tightly coupled between photosynthetic and calcification sinks (Bach, et al. 2013). On geological timescales, the extensive proliferation of *Coccolithophores* during the *Cretaceous* period and their efficient carbon fixation–calcification processes are thought to have driven large-scale carbonate deposition and significantly influenced atmospheric CO_2 and climate evolution, underscoring the long-term geobiological significance of coccolithophore CCMs.

At the cellular level, *Coccolithophore* CCMs rely on membrane transporters mediating CO_2/ HCO_3^- uptake and on multiple CA isoforms with distinct subcellular localizations. Genomic and transcriptomic analyses reveal CA variants associated with extracellular/periplasmic regions, cytosol, chloroplasts, nuclei, and coccolith vesicles,

each contributing to specific steps of DIC capture, conversion, and targeted delivery (Jensen, et al. 2020).

In addition, *Coccolithophores* express diverse ion transporters, proton pumps, and proteins influencing CO_2 membrane permeability, which collectively enable directed DIC transport and redistribution among subcellular compartments in response to environmental fluctuations (Blanco-Ameijeiras, et al. 2020). Calcification is intrinsically coupled to proton production or consumption, necessitating tight control of vesicular pH and DIC speciation. Regulation of CA activity and ion transport maintains optimal conditions within the coccolith vesicle and dynamically balances carbon allocation between photosynthesis and calcification, a defining feature of coccolithophore CCMs (Faucher, et al. 2025).

3. Diversity and evolution of carbonic anhydrase classes in microalgae

α-Carbonic anhydrases (α-CAs) are predominantly found in green algae and secondary endosymbiotic lineages. They typically adopt monomeric or dimeric structures, harbor Zn^{2+} at the active site, and exhibit the highest catalytic turnover rates among CA classes. α-CAs are commonly localized to extracellular spaces or chloroplast membranes, where they facilitate rapid CO_2 generation for downstream fixation (DiMario, et al. 2017).

β-Carbonic anhydrases (β-CAs) are widely distributed in cyanobacteria and eukaryotic microalgae. These enzymes usually form dimers or higher-order oligomers and play a key role in stabilizing stromal HCO_3^- pools. In both chloroplasts and carboxysomes, β-CAs provide substrate buffering for lumenal or shell-associated α-CAs, thereby contributing to efficient CO_2 supply to Rubisco (de Oliveira Maciel, et al. 2022).

γ-Carbonic anhydrases (γ-CAs) occur in cyanobacteria and some eukaryotic algae and typically form trimeric complexes. Their active sites may coordinate Zn, Fe, or Co, enabling functional flexibility under hypoxic or extreme environmental conditions. In some systems, γ-CAs have also been implicated in links between carbon metabolism and energy conversion (Cainzos, et al. 2021).

δ- and ζ-Carbonic anhydrases are largely restricted to diatoms and represent lineage-specific adaptations. δ-CAs are generally associated with intermembrane regions of complex plastids, whereas ζ-CAs are Cd/Zn-dependent enzymes that enable carbon acquisition under Zn-limited marine conditions, reflecting a biochemical

response to trace metal scarcity in oceanic environments (Alterio, et al. 2021).

η-, θ-, and ι-Carbonic anhydrases have been identified in parasitic protists or marine microalgae and remain comparatively less characterized. These enzymes often exhibit unique metal dependencies (e.g., Mn^{2+} or metal-independent activity) and specialized subcellular localization patterns, suggesting roles in localized CO_2 enrichment and microenvironmental regulation rather than bulk carbon flux control (Nawaly, et al. 2023).

***Table S1** Basic characteristics of various types of CA related to microalgae*

Class	Main Distribution	Metal Center	Oligomeric State	Catalytic Activity	Function in Algae / Photosynthetic Organisms	References
α	Animals, green algae, marine microalgae	Zn^{2+}	Monomer / Dimer	Highest	Core CCM component; supplies CO_2 to Rubisco; catalyzes ester/thioester hydrolysis	(Lee, et al. 2018)
β	Plants, cyanobacteria, green algae	Zn^{2+}	Dimer, Tetramer, Octamer	Moderate high	Stromal and carboxysomal CA; stabilizes carbon supply for Calvin cycle	(de Oliveira Maciel, et al. 2022)
γ	Archaea, cyanobacteria	Zn / Fe / Co	Trimer	Low	Adaptation to extreme environments; some isoforms linked to energy metabolism	(Cainzos, et al. 2021)
δ	Diatoms	Zn / Ca	Multimer	Moderate	Compensatory mechanism under Zn-limited seawater conditions	(Alterio, et al. 2021)
ζ	Diatoms	Ca^{2+}	Multimer	Low-moderate	Ca^{2+}-dependent CA; adaptation to Zn deficiency	(Alterio, et al. 2021)
η	Plasmodium	Zn^{2+}	Monomer	Low	Parasite metabolism (non-photosynthetic); catalyzes ester/thioester hydrolysis; first enzyme in pyrimidine synthesis	(Giovannuzzi, et al. 2022)
θ	Marine microalgae	Zn^{2+}	Diverse	Not fully characterized	Key regulator of multi-layered chloroplast CCM; catalyzes ester/thioester hydrolysis	(Nawaly, et al. 2023)
ι	Marine microalgae	Mn^{2+} / free	Not fully characterized	Unknown	Compensatory CA under low-metal conditions; catalyzes ester/thioester hydrolysis	(Hirakawa, et al. 2021)

4. Evolutionary origin and major forms of the Rubisco superfamily (Forms I–IV)

Rubisco originated ~3.5–4.0 billion years ago in an anoxic, CO_2-rich early Earth, where ancestral proteins likely functioned in non-photosynthetic metabolism (Taylor-Kearney, et al. 2024). Based on sequence, structure, and function, the Rubisco superfamily is divided into four major forms (Forms I–IV), reflecting adaptation to the atmospheric transition from high CO_2/no O_2 to low CO_2/high O_2 (Taylor-Kearney, et al. 2024). Form IV (Rubisco-like proteins, RLPs) represents the most ancient lineage, widespread in archaea and bacteria, catalyzing reactions in methionine salvage or other enediol pathways, without CO_2 fixation activity (Tabita, et al. 2008). Form III is predominantly found in anaerobic archaea (e.g., methanogens), participating in C_1 metabolism and representing one of the earliest RuBP-associated catalytic branches (Frolov, et al. 2019). Form II emerged ~3.5 Ga in certain bacteria (e.g., purple bacteria), typically as L_2 dimers (occasionally L_6), capable of carboxylation but highly O_2-sensitive with low CO_2/O_2 specificity (Frolov, et al. 2019). Form I evolved ~2.5–3.0 Ga around the Great Oxidation Event; incorporation of small subunits (SSU) produced the L_8S_8 hexadecamer, markedly improving CO_2/O_2 specificity, and establishing the dominant form in modern oxygenic photosynthesis (Davidi, et al. 2020). Form I is further subdivided into IA (marine cyanobacteria), IB (green algae and land plants), IC (some bacteria), and ID (red algal lineage), exhibiting systematic trade-offs among $K_{cat}C$, K_C, and $S_{C/O}$ (Capó-Bauçà, et al. 2024).

Rubisco catalytic performance in microalgae is primarily defined by three kinetic parameters: the carboxylation turnover rate ($K_{cat}C$), the Michaelis constant for CO_2 (K_C

or K_mCO_2), and the CO_2/O_2 specificity factor ($S_{C/O}$). Extensive comparative analyses support an evolutionary trade-off among these parameters, whereby higher $K_{cat}C$ typically coincides with lower $S_{C/O}$ (Prywes, et al. 2025). The diversity of microalgal Rubisco arises from multiple endosymbiotic events and horizontal gene transfer. Cyanobacteria predominantly harbor Form IA or IB Rubisco, whereas eukaryotic microalgae are dominated by Form IB (green lineage) and Form ID (red lineage); a few dinoflagellates retain Form II (Prywes, et al. 2023). The evolutionary transition of Rubisco from an anaerobic metabolic enzyme to a photosynthetic core catalyst was driven by declining atmospheric CO_2 and rising O_2 levels (Prywes, et al. 2023).

5. Reference

Alterio, V., Langella, E., Buonanno, M., Esposito, D., Nocentini, A., Berrino, E., Bua, S., Polentarutti, M., Supuran, C.T., Monti, S.M. and De Simone, G. (2021). Zeta-carbonic anhydrases show CS2 hydrolase activity: A new metabolic carbon acquisition pathway in diatoms? *Computational and Structural Biotechnology Journal* 19, 3427-3436.

Bach, L.T., Mackinder, L.C.M., Schulz, K.G., Wheeler, G., Schroeder, D.C., Brownlee, C. and Riebesell, U. (2013). Dissecting the impact of CO_2 and pH on the mechanisms of photosynthesis and calcification in the coccolithophore Emiliania huxleyi. *New Phytologist* 199, 121-134.

Blanco-Ameijeiras, S., Stoll, H.M., Zhang, H. and Hopkinson, B.M. (2020). Influence of Temperature and CO_2 On Plasma-membrane Permeability to CO_2 and HCO_3^- in the Marine Haptophytes Emiliania huxleyi and *Calcidiscus leptoporus* (*Prymnesiophyceae*). *Journal of Phycology* 56, 1283-1294.

Cainzos, M., Marchetti, F., Popovich, C., Leonardi, P., Pagnussat, G. and Zabaleta, E. (2021). Gamma carbonic anhydrases are subunits of the mitochondrial complex I of diatoms. *Molecular Microbiology* 116, 109-125.

Capó-Bauçà, S., Iñiguez, C. and Galmés, J. (2024). The diversity and coevolution of Rubisco and CO_2 concentrating mechanisms in marine macrophytes. *New Phytologist* 241, 2353-2365.

Davidi, D., Shamshoum, M., Guo, Z., Bar-On, Y.M., Prywes, N., Oz, A., Jablonska, J., Flamholz, A., Wernick, D.G., Antonovsky, N., de Pins, B., Shachar, L.,

Hochhauser, D., Peleg, Y., Albeck, S., Sharon, I., Mueller-Cajar, O. and Milo, R. (2020). Highly active rubiscos discovered by systematic interrogation of natural sequence diversity. *The EMBO Journal* 39, EMBJ2019104081.

de Oliveira Maciel, A., Christakopoulos, P., Rova, U. and Antonopoulou, I. (2022). Carbonic anhydrase to boost CO_2 sequestration: Improving carbon capture utilization and storage (CCUS). *Chemosphere* 299, 134419.

DiMario, R.J., Clayton, H., Mukherjee, A., Ludwig, M. and Moroney, J.V. (2017). Plant Carbonic Anhydrases: Structures, Locations, Evolution, and Physiological Roles. *Molecular Plant* 10, 30-46.

Faucher, G., Haunost, M., Paul, A.J., Tietz, A.U.C. and Riebesell, U. (2025). Growth response of Emiliania huxleyi to ocean alkalinity enhancement. *Biogeosciences* 22, 405-415.

Frolov, E.N., Kublanov, I.V., Toshchakov, S.V., Lunev, E.A., Pimenov, N.V., Bonch-Osmolovskaya, E.A., Lebedinsky, A.V. and Chernyh, N.A. (2019). Form III RubisCO-mediated transaldolase variant of the Calvin cycle in a chemolithoautotrophic bacterium. *Proceedings of the National Academy of Sciences* 116, 18638-18646.

Giovannuzzi, S., De Luca, V., Nocentini, A., Capasso, C. and Supuran, C.T. (2022). Coumarins inhibit η-class carbonic anhydrase from Plasmodium falciparum. *Journal of Enzyme Inhibition and Medicinal Chemistry* 37, 680-685.

He, S., Crans, V.L. and Jonikas, M.C. (2023). The pyrenoid: the eukaryotic CO_2-concentrating organelle. *The Plant Cell* 35, 3236-3259.

Huffine, C.A., Zhao, R., Tang, Y.J. and Cameron, J.C. (2023). Role of carboxysomes in cyanobacterial CO_2 assimilation: CO_2 concentrating mechanisms and metabolon implications. *Environmental Microbiology* 25, 219-228.

Jensen, E.L., Maberly, S.C. and Gontero, B. (2020). Insights on the Functions and Ecophysiological Relevance of the Diverse Carbonic Anhydrases in Microalgae. *International Journal of Molecular Sciences* 21, 2922.

Kerfeld, C.A. and Melnicki, M.R. (2016). Assembly, function and evolution of cyanobacterial carboxysomes. *Current Opinion in Plant Biology* 31, 66-75.

Lee, S.-H., McIntyre, D., Honess, D., Hulikova, A., Pacheco-Torres, J., Cerdán, S., Swietach, P., Harris, A.L. and Griffiths, J.R. (2018). Carbonic anhydrase IX is a pH-stat that sets an acidic tumour extracellular pH in vivo. *British Journal of Cancer* 119, 622-630.

Meyer, M.T., Itakura, A.K., Patena, W., Wang, L., He, S., Emrich-Mills, T., Lau, C.S., Yates, G., Mackinder, L.C.M. and Jonikas, M.C. (2020). Assembly of the algal CO_2-fixing organelle, the pyrenoid, is guided by a Rubisco-binding motif. *Science Advances* 6, eabd2408.

Nawaly, H., Tanaka, A., Toyoshima, Y., Tsuji, Y. and Matsuda, Y. (2023). Localization and characterization θ carbonic anhydrases in Thalassiosira pseudonana. *Photosynthesis Research* 156, 217-229.

Pierella Karlusich, J.J., Bowler, C. and Biswas, H. (2021). Carbon Dioxide Concentration Mechanisms in Natural Populations of Marine Diatoms: Insights From Tara Oceans. *Frontiers in Plant Science* 12, 657821.

Prywes, N., Phillips, N.R., Oltrogge, L.M., Lindner, S., Taylor-Kearney, L.J., Tsai, Y.-C.C., de Pins, B., Cowan, A.E., Chang, H.A., Wang, R.Z., Hall, L.N., Bellieny-Rabelo, D., Nisonoff, H.M., Weissman, R.F., Flamholz, A.I., Ding, D., Bhatt, A.Y., Mueller-Cajar, O., Shih, P.M., Milo, R. and Savage, D.F. (2025). A map of the rubisco biochemical landscape. *Nature* 638, 823-828.

Prywes, N., Phillips, N.R., Tuck, O.T., Valentin-Alvarado, L.E. and Savage, D.F. (2023). Rubisco Function, Evolution, and Engineering. *Annual Review of Biochemistry* 92, 385-410.

Tabita, F.R., Satagopan, S., Hanson, T.E., Kreel, N.E. and Scott, S.S. (2008). Distinct form I, II, III, and IV Rubisco proteins from the three kingdoms of life provide clues about Rubisco evolution and structure/function relationships. *Journal of Experimental Botany* 59, 1515-1524.

Taylor-Kearney, L.J., Wang, R.Z. and Shih, P.M. (2024). Evolution and origins of rubisco. *Current Biology* 34, R764-R767.

Trettel, D.S., Pacheco, S.L., Laskie, A.K. and Gonzalez-Esquer, C.R. (2024). Modeling bacterial microcompartment architectures for enhanced cyanobacterial carbon fixation. *Frontiers in Plant Science* 15, 1346759.

Shen, C., Dupont, C.L. and Hopkinson, B.M. (2017). The diversity of CO_2-concentrating mechanisms in marine diatoms as inferred from their genetic content. *Journal of Experimental Botany* 68, 3937-3948.
